# Annexin A2: A Double-Edged Sword in Pathogen Infection

**DOI:** 10.3390/pathogens13070564

**Published:** 2024-07-04

**Authors:** Tianyu Wang, Dengshuai Zhao, Yuanhang Zhang, Dixi Yu, Guoping Liu, Keshan Zhang

**Affiliations:** 1 Guangdong Provincial Key Laboratory of Animal Molecular Design and Precise Breeding, School of Life Science and Engineering, Foshan University, Foshan 528225, China; 2 College of Animal Science and Technology, Yangtze University, Jingzhou 434023, China

**Keywords:** annexin A2, inflammation, virus, bacteria

## Abstract

Annexin A2 (ANXA2) is a multifunctional calcium- and phospholipid-binding protein that plays an important role in various cells. During pathogen infections, ANXA2 modulates the nuclear factor kappa-B (NF-κB) and cell apoptosis signaling pathways and guides the chemotaxis of inflammatory cells toward inflammation sites, thereby protecting the host organism through the modulation of the inflammatory response. In addition, ANXA2 can regulate immune responses, and in certain pathogen infections, it can interact with pathogen proteins to facilitate their invasion and proliferation. This review provides an overview of the research progress on how ANXA2 regulates pathogen infections.

## 1. Introduction

Annexin A2 (ANXA2) is a pleiotropic calcium- and anionic-phospholipid-binding protein that exists as a monomer [1]. Furthermore, it can form a heterotetrameric complex with the fibrinogen receptor protein S100A10 (p11) [1], known as AIIT or A2t. Notably, the biochemical properties of the monomeric and heterotetrameric forms of ANXA2 are markedly different [2]. Interestingly, S100A10 cannot exist in the cells in the absence of ANXA2 [3]. Annexins are involved in several cellular processes, including cytophagy, cytotransmission, signal transduction, proliferation, invasion, and metastasis, thereby affecting tumor growth [4,5]. ANXA2 was initially identified as a substrate for the tyrosine kinase V-Src, a gene product of the sarcoma virus that promotes cell transformation [5]. Subsequently, the overexpression of ANXA2 was reported in various tumors, such as hepatocellular carcinoma (HCC) and breast cancer [6]. ANXA2 can affect the growth and development of tumor cells and regulate the migration, invasion, and adherence of tumor cells [7,8]. In addition, ANXA2 regulates inflammatory responses and plays an important role in the infection process of bacteria, viruses, and other pathogens [9,10,11].

## 2. Structure and Localization of Annexin A2

**Figure 1:**
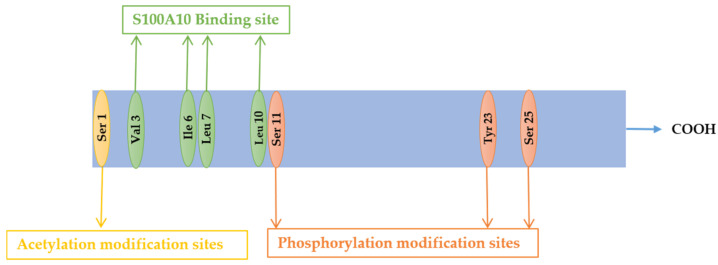
N-terminal structural domain of ANXA2. The N-terminus of ANXA2 contains one acetylation modification site (Ser 1), three phosphorylation modification sites (Ser 11, Tyr 23, and Ser 25), and the S100A10 binding site (Val 3, Ile 6, Leu 7, and Leu 10).

ANXA2 is a membrane-bound protein consisting of a variable amino acid-terminal structural domain, which includes sites for post-translational modification and protein–protein interactions, and a homologous carboxylic structural domain [1]. The carboxyl core region contains four repeating 70-amino-acid fragments, with each fragment consisting of five α-helices (A–E), four of which are antiparallel, and the fifth is perpendicular to them [12], forming a right-handed superhelix. Furthermore, the carboxy-terminal core region includes binding sites for calcium ions, anionic phospholipids, heparin, DNA, and F-actin [13,14]. ANXA2 consists of 339 amino acids and contains an amphipathic α-helical region at its amino acid terminus, with a hydrophobic surface that binds to S100A10 [1]. The N-terminal structural domain also includes serine and tyrosine phosphorylation sites [15], a reactive hemicysteine [16], and exonucleic acid sequences [17]. The N-terminal structural domain consists of thirty amino acid residues, one acetylation site, Ser1, three phosphorylation sites (Ser11, Ser25, and Tyr23), and a binding site for S100A10 (Figure 1).

ANXA2 is widely found in several mammalian cell types, including monocytes, macrophages, and endothelial and myeloid cells [18]. ANXA2 tetramers are present in the cytoskeleton beneath the membrane in various cells and are involved in several membrane-associated events, such as the calcium-dependent regulation of cytotoxicity [19]. The ANXA2 monomer is found predominantly in the cytosol of various eukaryotic organisms, with a small fraction located in the nucleus [19].

## 3. Mechanisms through Which Annexin A2 Regulates Pathogen Infection

### 3.1. Annexin A2 Activates Epithelial–Mesenchymal Transition and Promotes Tumor Cell Invasion and Metastasis

The epithelial–mesenchymal transition (EMT) is the conversion of epithelial cells into mesenchymal cells during wound healing, tissue regeneration, organ fibrosis, and tumor progression [20]. The EMT enhances the motility and invasiveness of tumor cells [20]. Studies on breast cancer have shown that ANXA2 expression is strongly correlated with epidermal growth factor (EGFR) expression and the EMT. The knockdown of ANXA2 inhibits the EMT via the autophosphorylation and activation of EGF upon binding to EGFR and the phosphorylation of Tyr23 in the N-terminal structural domain of ANXA2 [21]. Phosphorylated ANXA2 induces the phosphorylation of STAT3, which translocates from the cytoplasm to the nucleus and upregulates the expression of the transcription factor Slug, which promotes the EMT [21]. In another study, the ANXA2 antibody was found to inhibit EGF-induced homodimerization, phosphorylation, and the intrinsic action of EGFR [22]. This suggests that a reduction in ANXA2 can suppress the phosphorylation level of EGF with EGFR, thereby inhibiting the EMT. Therefore, the loss of ANXA2 might be a crucial therapeutic strategy to inhibit the EMT, offering a novel direction for disease treatment.

**Figure 2:**
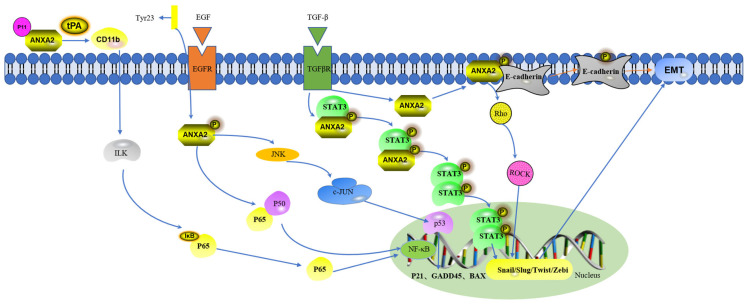
Selected signaling pathways involved in ANXA2.

Studies on rectal cancer have shown that the cytokine TGF-β, an inducer of the EMT, can trigger the upregulation and phosphorylation of ANXA2 [23], which in turn induces the internalization of the E-cadherin protein. This causes the phosphorylation of the transcription factor STAT3, which migrates to the nucleus and induces the expression of the transcription factor Slug, inhibiting the transcription of the CDH1 gene [23]. The downregulation of the CDH1 gene, which serves as a mediator of a homotypic calcium-dependent adhesion molecule that mediates cell junctions, promotes the EMT [23] (Figure 2). This suggests that the onset of ANXA2 phosphorylation plays an important regulatory role in the EMT process. Studies on HCC showed that cancer-associated mesenchymal stem cells (MSCs) contribute to the development and metastasis of HCC [24]. Mechanistic studies have demonstrated that long-stranded noncoding RNA-MUF, an MSC upregulation factor, can interact with ANXA2, which activates the Wnt/β-catenin signaling pathway and the EMT [24]. The participation of ANXA2 activates the EMT, promoting cell invasion and migration. This further emphasizes the crucial role of ANXA2 in facilitating disease progression.

By reducing ANXA2 expression in bladder cancer T24 cells using RNA interference (RNAi), the authors found that knocking down the ANXA2 gene significantly inhibited the proliferation, migration, and invasion of T24 cells [25]. ANXA2 was isolated from the serum of aggressive triple-negative breast cancer (TNBC) patients, and high levels of ANXA2 protein or mRNA were found to correlate with the degree of tumor malignancy. Furthermore, patients with low levels of ANXA2 protein or mRNA did not respond to chemotherapy, and patients with high levels of ANXA2 protein or mRNA did respond to chemotherapy, suggesting that ANXA2 protein or mRNA may serve as a combined predictive biomarker for chemotherapy response in aggressive TNBC patients [26].

### 3.2. Annexin A2 Is Involved in the Regulation of Innate Immune Responses

Inflammation is triggered when innate immune cells detect infection or tissue injury [27]. In a study using a mouse model of *Klebsiella pneumoniae*, mice with reduced ANXA2 expression eventually died due to excessive oxidative stress after being infected with bacteria, whereas all mice with normal ANXA2 expression survived [28]. A significant increase in the levels of pro-inflammatory cytokines was observed in the mice with negative ANXA2 expression, suggesting that ANXA2 can regulate inflammation by promoting anti-inflammatory signaling [28]. In studies on cryptococcal infections, the macrophages in ANXA2-deficient mice were found to phagocytose yeast cells less efficiently than those in mice with a normal expression of ANXA2 [29]. Therefore, these ANXA2-deficient mice had a higher mortality rate than normal mice due to the dysregulation of inflammatory responses [29]. This suggests that ANXA2 protects the host from damage due to excessive inflammatory response during bacterial infection. A mouse study proved that ANXA2 can act as an adhesion receptor for rickettsiae on the endothelial surface in rickettsial infections [30]. In the absence of ANXA2, the adhesion of Rickettsia to vascular endothelial cells is decreased, thereby increasing the number of bacteria in the bloodstream [30]. Furthermore, ANXA2 regulates the inflammatory response in bacterial infections and autophagy through the Akt1-mTOR-ULK1/2 signaling pathway during *Pseudomonas aeruginosa* infections, which promotes host immunity against bacteria [31]. These studies demonstrate that ANXA2 modulates the inflammatory response and autophagy during bacterial and fungal infection, promoting the body’s immune response against bacteria.

Nuclear factor kappa-B (NF-κB), a critical transcription factor in the inflammatory response, mediates inflammation [32]. The ANXA2 tetramer induces the phosphorylation of various kinases in the MAPK pathway and induces the translocation of NF-κB P65, which stimulates the production of inflammatory mediators, such as IL-1β, IL-6, and TNF-α, in human macrophages [33]. In studies investigating the mechanism by which tissue-type plasminogen activator (tPA) regulates the NF-κB pathway, tPA was found to induce ANXA2 to aggregate and interact with the integrin CD11b, thereby activating the integrin-linked kinase signaling pathway (ILK) [34]. The activation of this pathway induces the degradation and phosphorylation of IκB, which promotes NF-κB activity [34] (Figure 2). Heterotetramers composed of ANXA2 and S100A10 are also important for mediating inflammation and the fibronectin-activated NF-κB signaling pathway: the deletion of this heterotetramer inhibits the production of inflammatory cytokines, such as TNF-α and IL-6, by macrophages [35]. In lipopolysaccharide (LPS)-induced inflammatory responses, S100A10 deficiency significantly reduces the production of cytokines, such as TNF-α, IL-1β, and IL-10, and inhibits LPS-induced activation of MAPKs [36]. In gemcitabine-resistant pancreatic cancer, ANXA2 could interact directly with P50 and cotranslocate into the nucleus, thereby modulating NF-κB signaling [37]. This upregulated the genes encoding IL-6, which induced gemcitabine resistance in pancreatic cancer, suggesting that ANXA2 might be a useful therapeutic target [37]. Furthermore, ANXA2 promotes cisplatin resistance by inhibiting P53 expression in cells through the activation of the JNK/c-Jun signaling pathway, which reduces the expression of the P53-regulated apoptotic genes *p21*, *GADD45*, and *BAX* [38] (Figure 2). In non-small-cell lung cancer cell model studies, phosphorylated ANXA2 activated JNK, thereby inhibiting P53 expression at the transcriptional level and mediating c-JUN stabilization [39]. These results demonstrate that ANXA2 can regulate NF-κB through several pathways, thereby participating in the regulation of inflammatory responses and apoptosis.

In addition to its role in *Klebsiella pneumoniae*, *Pseudomonas aeruginosa* [28,30,31], and rickettsiae infection, ANXA2 contributes to viral pathogenesis [40,41,42,43]. ANXA2 has been shown to interact with several viral proteins, including NS1 of the avian influenza virus (IAV) H5N1, US3 of the pseudorabies virus (PRV), E2 glycoprotein of the classical swine fever virus (CSFV), and Nsp9 of the porcine reproductive and respiratory syndrome virus (PRRSV) [40,41,42,43]. All these interactions can contribute to the development of viral infections. Using yeast two-hybrid techniques, the authors found that during enterovirus 71 (EV71) infection, the VP1 protein of EV71 binds to ANXA2 on the cell surface, facilitating viral entry into the cell [44]. Viral life cycles consist of three main phases: (1) attachment and entry, (2) genome replication and expression, and (3) assembly, maturation, and egress [45]. ANXA2 participates in all these three major stages in different viruses [45], indicating its role in natural antiviral immunity at different stages after viral infection and thereby promoting the growth of viruses within the host.

Furthermore, ANXA2 can enable the chemotaxis of the monocyte, macrophage, and neutrophil to the inflammation site [46,47,48]. ANXA2 promotes cytokine-directed monocyte or macrophage migration through the extracellular matrix and the mobilization of monocytes to the inflammation site [47]. The interaction of ANXA2 with CD44 in the presence of complement-activated serum and vitamin D structural proteins also promotes neutrophil chemotaxis, whereas ANXA2 knockdown or the use of an anti-ANXA2 antibody inhibits this chemotactic effect on monocytes and neutrophils [46,47,48], demonstrating that ANXA2 is involved in the chemotaxis of inflammatory cells.

### 3.3. Annexin A2 Regulates Adaptive Immunity

In addition to its role in innate immunity, ANXA2 acts as a self-antigen in regulating adaptive immunity [49,50,51,52]. In vitro experiments demonstrated that ANXA2 is the antigen of serum IgG from asbestos-exposed lung cancer patients by serological analysis of a recombinantly expressed cDNA clone technology and is overexpressed in cancer and normal tissues [49]. Moreover, serum containing overexpressed ANXA2 showed higher levels of IL-6; the siRNA-mediated inhibition of ANXA2 expression in prostate cancer cells reduced IL-6 secretion, while the restoration of ANXA2 expression by transfection of the ANXA2 gene normalized IL-6 secretion [49]. This indicates a close correlation between ANXA2 and IL-6 [49]. In autoimmune diseases such as antiphospholipid syndrome, Behcet’s disease, and lupus nephritis [50,51,52], ANXA2 participates in disease development by acting as a self-antigen. The authors used the serum of pancreatic ductal adenocarcinoma (PDAC) patients for the identification of expressed proteins, and the ANXA2 protein was identified by mass spectrometry [53]. In mouse model studies, immunotherapy targeting ANXA2, in combination with anti-PD-1 antibodies, resulted in a significant increase in IFNγ expression in T cells targeting ANXA2, thereby alleviating the disease in a PDAC model [54].

ARG1, an arginase, allows myeloid cells to consume arginine and suppresses the survival of T cells [55]. ANXA2 induced ARG1 mRNA expression through the TLR2/MYD88 axis in neutrophils in a mouse model. Hence, in a mouse model, ANXA2 can inhibit lymphocyte function by regulating ARG1 expression [56]. Dendritic cells (DCs) are antigen-presenting cells that play a crucial role in nasopharyngeal carcinoma [57]. In nasopharyngeal carcinoma, the interaction between tumor cells and ANXA2 can activate dendritic-cell-specific ICAM-grabbing non-integrin (DC-SIGN), also known as CD209 [57]. In addition, ANXA2 can bind to TLR2 and promote DC maturation and cross-priming in a mouse model [58]. Moreover, in mouse splenocytes, the binding of ANXA2 to TLR2 can upregulate the expression of CD80 and CD86, enhancing the antigen-specific immune response of T cells and inducing the secretion of IL-12, tumor necrosis factor-α (TNF-α), and IFN-γ [58]. These findings illustrate that ANXA2 can regulate adaptive immune responses by modulating immune cells and cytokines, emphasizing its crucial role in the immune system.

## 4. Annexin A2 and Bacterial Infection

ANXA2 is involved in infection by various bacteria, such as Rickettsia, *Escherichia coli*, and *Salmonella* [30,59,60]. These bacteria can use ANXA2 for their own growth and invasion. Rickettsia primarily infects endothelial cells (ECs), and studies using atomic force microscopy showed that ANXA2 on the surface of brain microvascular endothelial cells (BMECs) acts as an adhesion receptor for Rickettsia [30]. Rickettsial adhesin outer membrane protein B (OmpB) serves as the ligand for ANXA2, and the absence of ANXA2 impedes Rickettsia’s attachment to ECs and the vascular surface in the C57BL/6 mouse–R. australis model [30]. Treatment with anti-ANXA2 antibodies has been shown to reduce surface-associated *Staphylococcus aureus* on human umbilical vein endothelial cells (HUVECs) [30]. In addition, cAMP directly activates the exchange protein directly activated by cAMP 1 (EPAC1) and regulates Rickettsia adhesion and invasion of human umbilical vein endothelial cells (HUVECs) [61]. The binding strength between Rickettsia’s OmpB and EPAC1 on the surface of live mouse brain microvascular ECs (BMECs) depends on the phosphorylation of ANXA2 at Tyr23 [62]. This binding strength is associated with ANXA2 receptor phosphorylation, indicating that EPAC1 regulates Rickettsia adhesion by phosphorylating ANXA2 and binding to OmpB [62].

In vitro mammalian cell experiments showed that Mycoplasma pneumoniae can synthesize a cytotoxin known as community-acquired respiratory distress syndrome (CARDS) toxin, which binds to the human pulmonary surfactant protein A (SP-A) [63]. Immunofluorescence analysis revealed that this toxin colocalized with ANXA2 on the human A549 airway cells’ surface and within the human A549 airway cells [64]. In A549 cells, the binding specificity between the CARDS toxin and ANXA2 occurs via the carboxyl-terminal end of the CARDS toxin [64]. The inhibition of ANXA2 reduces the binding and internalization of the CARDS toxin in the A549 cells [64]. In addition, in in vitro experiments, the GroEL protein (heat shock protein 60) of *Mycoplasma bovis* induces apoptosis in peripheral mononuclear cells (PMCs) [65]. Studies exploring the effects of the GroEL protein on cell apoptosis revealed an interaction between ANXA2 and the GroEL protein [66]. The stimulation of PMCs with GroEL upregulates the apoptosis factors Bax/Bcl2 and caspase, accompanied by a significant increase in ANXA2 expression, indicating that GroEL in *Mycoplasma gallisepticum* can interact with ANXA2 to induce cell apoptosis [66]. Furthermore, ANXA2 expression was upregulated in *Mycoplasma bovis*-infected bovine lung epithelial cells (EBL) [67]. Treatment with ANXA2 antibodies reduces the adhesion of *Mycoplasma bovis* to EBL cells, and the transcription of IL-8 and CXCL5 as well as the phosphorylation activity of NF-κB and MAPK in the cells significantly increase [67]. This suggests that *Mycoplasma bovis* can use ANXA2 to promote its infection and influence the cow’s inflammatory response [67]. In addition, a study found that the lipoprotein LppA from *Mycoplasma bovis* interacted with ANXA2 by IP-MS and CO-IP methods, and LppA was able to promote the enrichment of ANXA2 on the embryonic bovine lung (EBL) cell membranes, demonstrating that LppA is able to promote the adhesion of *M. bovis* to EBL cells by interacting with ANXA2 [68]. The above studies suggest that *mycoplasmas* may use ANXA2 to mediate their adhesion and invasion, thereby influencing the inflammatory response and promoting mycoplasma infection.

The invasion of enteropathogenic *Salmonella* relies on the type III secretion system (T3SS), and the T3SS effector SopB is a dual-functional protein that requires the recruitment of ANXA2 and the giant phosphoprotein AHNAK to the invasion site in the MDCK cells and the HeLa cells. This recruitment occurs in a SopB-dependent manner and is a crucial component of *Salmonella* invasion [59]; SopB is an inositol phosphatase [69]. Additionally, AHNAK can directly bind to ANXA2 through interaction with P11, which serves as a scaffold that connects actin remodeling and signaling pathways [59]. In enterohemorrhagic *Escherichia coli*, EspL2, an effector delivered by the type III secretion system, directly binds to ANXA2 clustered with F-actin in the COS-7 cells and enhances its ability to aggregate F-actin [60]. This, in turn, promotes bacterial invasion by altering the morphology of the host cell membrane [60].

*Cryptococcus neoformans*, a fungus causing cryptococcal meningitis, invades the central nervous system (CNS) by penetrating the brain ECs, also known as the blood–brain barrier (BBB) [70,71]. It was shown that in hCMEC/D3 cells, *C. neoformans* can use signaling pathways and cellular cytoskeleton remodeling through ANXA2, S100A10, smooth muscle, and myosin to facilitate its traversal across the BBB, leading to cell necrosis [72]. In an in vitro model of the human BBB, it can also breach the BBB via a fungal metalloproteinase Mpr1, which promotes the binding of fungal cells to the BMECs [73]. Subsequent research demonstrated that the interaction between the Mpr1 protein and ANXA2 enabled the fungus to traverse the BBB. Mpr1 might promote the remodeling of the cytoskeleton in the hCMEC/D cells via ANXA2, thereby facilitating the transport of *C. neoformans* across the BBB [73]. Moreover, there is some evidence from in vitro and in vivo studies that ANXA2 controls *C. neoformans* infection by regulating macrophage function and influencing fungal morphology [29]. In vitro MBMEC cell line bEND.3 experiments demonstrated that the inhibition of ANXA2 significantly downregulates its partner protein S100A10 and significantly decreases the transversal efficiency of *C. neoformans* in the BMECs [74].

Using a proteomic approach, a study found that 325 proteins were upregulated when Staphylococcus aureus (*S. aureus*) infected bovine mammary epithelial MAC-T cells, including ANXA2 [75]. In another study on the ability of As to prevent *S. aureus*, As was found to reduce the expression of the *S. aureus* clumping factor (ClfB) and block its interaction with ANXA2, thereby reducing bacterial adherence and the levels of pro-inflammatory cytokines released during the infection of MAC-T cells [76]. In a study of Streptococcus anginosus (*S. anginosus*), the authors employed glutathione-S-transferase (GST) pull-down and co-immunoprecipitation (CO-IP) techniques to demonstrate that in Ges-1 and AGS cells the *S. anginosus* lipoprotein TMPC and ANXA2 facilitate *S. anginosus*-induced mitogen-activated protein kinase (MAPK) activation and contribute to its pro-tumorigenic role in the gastric epithelium [77].

Furthermore, surprisingly, ANXA2 has unexpected effects on sepsis due to severe symptoms caused by bacterial infections [78]. In cecal ligation and puncture (CLP) sepsis models, ANXA2 can suppress the inflammatory response in sepsis by regulating reactive oxygen species (ROS) and the IL-17 signaling pathway. The inhibition of ANXA2 results in a significant increase in ROS and IL-17, accompanied by increased pro-inflammatory cytokines and neutrophil infiltration in mouse colon tissues [78]. This highlights the beneficial aspect of ANXA2 in inhibiting the inflammatory response during sepsis [78].

## 5. Annexin A2 Protein and Viral Infections

ANXA2 is involved in the pathogenesis of viral infections in humans and animals as well as zoonotic diseases (Table 1).

### 5.1. Annexin A2 Regulates Cytomegalovirus Infection

Cytomegalovirus (CMV) is a DNA virus of the herpesvirus group that can be transmitted via infection or congenital transmission [95]. It causes several serious illnesses, including pneumonia and hearing loss [95]. Early identification of ANXA2 on the surface of CMV particles in human fibroblasts and the use of ANXA2 antibodies to bind to ANXA2 decreased the infectiousness of CMV in human foreskin fibroblast cell lines, suggesting that ANXA2 contributes to the entry of CMV into the cell and enhances viral infectivity [79,80]. In addition, endogenous ANXA2 promotes CMV infection, and in turn, CMV infection promotes the expression of ANXA2, which can activate human γδ T cells by recognizing cellular receptors, thereby inducing an inflammatory response [81,82,83]. These findings suggest that ANXA2 plays an important role in CMV infection and transport.

### 5.2. Annexin A2 Regulates Influenza Virus Replication

IAVs are highly contagious viruses comprising four types, of which influenza A and B viruses cause seasonal illnesses and epidemics of respiratory infections [96]. The replication of IAV type H1N1 in the Madin–Darby canine kidney (MDCK) cell line and IAV type H5N1 in the human lung epithelial A549 cell line was shown to be associated with ANXA2 [40,84]. ANXA2 and A2t, a fibrinogen receptor responsible for fibrinogen activation, were found on the envelope of IAV H1N1 viruses purified from culture supernatants of infected MDCK cells [84]. Furthermore, in MDCK cells, ANXA2-mediated fibrinogen activation promotes influenza virus replication [84]. In addition, the siRNA-mediated inhibition of ANXA2 suppressed the expression of viral proteins in the A549 cells and decreased the titer of viral progeny. Meanwhile, in the A549 cells, the overexpression of ANXA2 significantly increased the titer of IAV H5N1, demonstrating its role in IAV H5N1 replication [40] and its contribution to the development of influenza virus infection. By utilizing RNA interference (RNAi), the authors found that the ANXA2 protein is involved in the growth cycle of HPAI H7N9 in A549 cells, with the presence of hemagglutinin (HA), neuraminidase (NA), nucleoprotein (NP), and nucleoprotein acidic (PA) proteins that interact with HPAI H7N9 [85].

### 5.3. Annexin A2 Regulates Hepatitis C Virus Assembly and Replication

HCV is an enveloped, single positive-stranded RNA virus that contains six partial nonstructural proteins (NS2, NS3, NS4A, NS4B, NS5A, and NS5B) [97]. Although the nonstructural proteins are not present in the viral particles, mass spectrometry analysis after the immunoprecipitation of NS3/NS4A revealed that ANXA2 interacts with these nonstructural proteins [86]. Although the ANXA2 monomer colocalized with HCV NS proteins, its knockdown did not directly affect HCV RNA replication in Huh-7/Lunet cells but decreased viral titers inside and outside the cell [98]. Therefore, ANXA2 is probably involved in viral assembly rather than replication [98]. Immunofluorescence and electron microscopy analysis revealed that ANXA2 localized in the HCV replication complex in Huh7.5 cells and that NS4B, NS5A, and NS5B interacted with ANXA2 [87]. Further, the knockdown of ANXA2 in Huh7.5 cells via siRNA results in a reduction in the membrane network and RNA replication in HCV [87]. In contrast, the overexpression of ANXA2 in the HEK293 cells resulted in the enrichment of HCV NS proteins, thereby promoting HCV replication, demonstrating that ANXA2 has an important role in HCV replication [87].

### 5.4. Annexin A2 Regulates Pseudorabies Virus Replication

PRV is an enveloped DNA virus belonging to the Herpesviridae family [99]. It has been responsible for causing significant economic losses to the pork industry for several years [100]. *US3*, a key virulence gene of the herpes simplex virus, interferes with the defense mechanisms and prevents apoptosis in HeLa cells [101]. In PK-15 cells, US3 also interacts with ANXA2 to promote pseudorabies virus proliferation, while the knockdown of ANXA2 in 3D4/21 cells significantly inhibited PRV replication [41]. In addition, US3 induces the translocation of ANXA2 to interact with it extracellularly in PK-15 cells, as revealed by confocal microscopy [41]. The use of inhibitors targeting ANXA2 and Src kinase significantly inhibited PRV replication in PK-15 cells and mice and attenuated virus-induced organismal damage, suggesting that the inhibition of ANXA2 is a potential therapeutic approach for PRV [41].

### 5.5. Annexin A2 Regulates Porcine Reproductive and Respiratory Syndrome Virus Replication

PRRS is an infectious disease caused by PRRSV, also known as the “blue ear disease” which causes reproductive and respiratory disease in sows, resulting in significant losses to the pig industry each year [102]. The knockdown of ANXA2 using siRNA inhibits PRRSV replication in Marc-145 cells [88]. ANXA2 interacts with vimentin in PAMs, Marc-145 cells, and 293T cells, as revealed by confocal microscopy and co-immunoprecipitation, and this interaction promotes PRRSV replication [88]. NSP9, a nonstructural protein of PRRSV and an RNA-dependent RNA polymerase, is integral for virus–host protein (annexin A2, the zinc-finger antiviral protein, DEAD-Box Helicase 5) interactions during viral infections [42,103,104,105]. After PRRSV infection of Marc-145 cells, co-immunoprecipitation showed that NSP9 appears to interact with ANXA2 to promote viral replication [42]. The knockdown of ANXA2 in Marc-145 cells via siRNA interrupts this promotion and inhibits viral replication [42]. The use of AMG487, an inhibitor of CXCR3, significantly reduced the gene copy number of PRRSV and attenuated porcine lung injury [89]. Further, treatment with AMG487 significantly reduced ANXA2 expression and inhibited PRRSV replication in porcine macrophages [89]. This suggests that ANXA2 can promote PRRSV replication by interacting with cellular or viral proteins.

### 5.6. Annexin A2 Regulates Replication, Assembly, and Release of Classical Swine Fever

CSFV is an enveloped positive-stranded RNA virus whose genome consists of a 5′UTR, an ORF, and a 3′UTR encoding several proteins, including structural proteins (C, Erns, E1, and E2) and nonstructural proteins (N^pro^, P7, NS2, NS3, NS4A, NS4B, NS5A, and NS5) [106,107]. Among them, E2, the main envelope protein on the surface of swine fever virus particles, plays a key role in virulence, entry into host cells, and the immune response to the virus [108]. In PK-15 cells, while the siRNA-mediated knockdown of ANXA2 did not affect CSFV replication, it significantly reduced its production, suggesting that ANXA2 participates in the assembly and release of this virus [90]. In another study, the authors showed that ANXA2 had a greater effect on virus particle production than on CSFV replication and that knockdown using siRNA and overexpression mediated by the plasmid transfection of ANXA2 in PK-15 cells inhibited and promoted CSFV, respectively [43]. In addition, confocal microscopy and co-immunoprecipitation showed that the E2 and NS5A proteins of CSFV were able to bind ANXA2 in PK-15 cells [43,90]. The overexpression of E2 by plasmid transfection in PK-15 cells resulted in the upregulation of ANXA2 expression and promoted CSFV proliferation [43]. However, Western blot experiments showed that the replication of CSFV in PK-15 cells was significantly inhibited after treatment with an ANXA2-specific polyclonal antibody, suggesting that ANXA2 promotes viral replication during CSFV infection by binding to the E2 protein [43]. In addition, the disruption of the binding activity of NS5A to ANXA2 by constructing a mutant of the NS5A was found to significantly decrease the viral yield by tissue culture infective dose 50% (TCID_50_), but the Western blot results showed that it did not affect viral replication, suggesting that ANXA2 may enhance viral yield by binding NS5A [90]. These studies suggest that ANXA2 facilitates the replication and packaging release of CSFV and provide theoretical support for ANXA2 as a new cellular target for antiswine-fever therapy.

### 5.7. Annexin A2 Regulates Human Papillomavirus Internalization and Infection

HPV, a double-stranded DNA virus, can cause various benign or malignant tumors [109,110]. The viral particle comprises a major coat protein, L1, and a minor coat protein, L2 [110]. The heterotetrameric form of ANXA2 (A2t) binds to the epithelial cells containing HPV16 particles via the L2 minor proteins, promoting HPV16 infection [91]. Meanwhile, in HaCaT cells, inhibiting A2t using ANXA2 antibodies or ANXA2 ligands significantly reduces HPV16 infection [91]. The shRNA-mediated downregulation of A2t in HeLa cells inhibits both viral capsid internalization and infection, demonstrating that the presence of A2t promotes HPV16 internalization and the infection of host epithelial cells [91]. In a follow-up study, the interaction between A2t and HPV16 particles in HaCaT cells was shown to facilitate viral entry into host cells, and the use of the ANXA2 antibody inhibited this viral internalization [92]. In contrast, the use of the S100A10 antibody in HaCaT cells inhibited infection in the late nucleus, suggesting that ANXA2 and S100A10 may have different roles [92]. The knockdown of A2t and S100A10 using siRNA in HaCaT cells revealed that the deletion of A2t significantly inhibited viral infection progression within cells, reduced coat shedding, and accelerated the lysosomal degradation of HPV viral particles [92]. These findings suggest that A2t, a central mediator in the intracellular transport and infection of HPV, has a key role in promoting viral infection [91,92]. Moreover, in HeLa cells, ANXA2 can form a complex with CD63, a known vector of HPV transmission [93,111]. This demonstrates that the ANXA2 monomer has an independent role in promoting the HPV16 infection of HeLa cells [93].

### 5.8. Annexin A2 and other Viruses

Avian reovirus (ARV) σC protein is a cell attachment protein [112]. In Vero and DF-1 cells, ARV σC protein was shown to interact with cell-surface ANXA2 and the adhesion G protein-coupled receptor Latrophilin-2 (ADGRL2) by the proximity ligation assay (PLA) technique, and the inhibition of ANXA2 using a high-affinity ANXA2/S100 A10 heterotetramer inhibitor, A2ti-1, significantly reduced viral loads in Vero and DF-1 cells [94]. In addition, in an in vitro study of the hepatitis B virus, the authors demonstrated that rs1883832 is a single-nucleotide polymorphism (SNP) of the CD40 protein and that its risk allele T, in combination with ANXA2, can inhibit the expression of the CD40 gene, thereby suppressing HBV replication and transcription [113].

## 6. Discussion

Overall, this paper demonstrates the importance of ANXA2 in pathogen infections by describing the signaling pathways in which ANXA2 is involved and its role in various pathogen infections. During the early stages of infection, ANXA2, as a cell surface receptor, facilitates the adhesion and internalization of bacteria and viruses, promoting their replication, assembly, and release, which is detrimental to the host organism. In addition, numerous studies have shown that the overexpression of ANXA2 promotes infection by most pathogens, whereas the inhibition of ANXA2 using antibodies or siRNAs reduces infection by pathogens. These findings suggest that inhibitors or antibodies to ANXA2 may serve as potential therapeutic agents for the treatment of these diseases.

In the inflammatory response, ANXA2 is able to activate the NF-κB signaling pathway, promote the production of inflammatory mediators, and move specific cells to the site of inflammation by chemotaxis, thereby regulating the inflammatory response and promoting organismal defense and repair. This regulatory effect is favorable to the health of the organism. In this case, ANXA2 is a double-edged sword for the health of the organism.

We have also summarized the cell/animal models used in some of the above studies (Table 2). It is easy to see that most of the research is still directed towards in vitro cellular experiments and that in vivo experiments have only been carried out in mouse models. In vitro studies have been carried out on cells from many different species, and the link between ANXA2 and pathogens has been found in all of these cells. Therefore, our hypothesis is that the effect of ANXA2 on pathogens is not species-specific. In addition, there is a lack of in vivo studies, especially in human patients. It is therefore uncertain whether these conclusions can be extrapolated to human patients. This may also serve as a future research direction.

## Figures and Tables

**Table 1:** Proteins interacting with ANXA2 in different viral infections and roles.

Virus	Genome	Interoperating Protein	Observations	References
CMV	dsDNA	--	-ANXA2 promotes viral infection -CMV can promote ANXA2 expression -ANXA2-mediated activation of fibrinogen promotes viral replication	[79,80,81,82,83]
IAV	ssRNA	NS1 (H5N1) and NP, PA (H7N9)	-ANXA2 interacts with NS1 protein in IAV H5N1 -ANXA2 overexpression increases the titer of IAV H5N1 -ANXA2 protein is involved in the growth cycle of HPAI H7N9	[40,84,85]
HCV	ssRNA	NS	-ANXA2 interacts with nonstructural (NS) proteins -ANXA2 promotes HCV assembly and replication	[86,87]
PRV	ssRNA	US3	-ANXA2 interacts with US3 -ANXA2 promotes PRV replication	[41]
PRRSV	ssRNA	NSP9	-ANXA2 interacts with vimentin -ANXA2 interacts with NSP9 -ANXA2 promotes PRRSV replication	[42,88,89]
CSFV	ssRNA	E2 and NS5A	-ANXA2 interacts with E2 -ANXA2 interacts with NS5A -ANXA2 promotes CSFV replication, assembly, and release	[43,90]
HPV	dsDNA	--	-A2t promotes HPV internalization and infection -Knockdown of ANXA2 suppresses HPV16 internalization	[91,92,93]
ARV	dsRNA	σC	Inhibition of ANXA2 leads to significant reduction in viral load	[94]

**Table 2:** Cell/animal models for the study of ANXA2 and pathogens.

Pathogens		In Vivo/Vitro	Cell/Animal Models	References
Bacterium	*Klebsiella pneumoniae*	vivo	Mice	[28]
	*Rickettsia*	vivo	Mice	[30]
		vitro	BMECs (mice) and HUVECs (human)	[30,61,62]
	*Staphylococcus aureus*	vitro	HUVECs (human) and MAC-T cells (human)	[30,75,76]
	*Mycoplasma pneumoniae*	vitro	A549 airway cells (human)	[64]
	*Mycoplasma bovis*	vitro	EBL (cow)	[67,68]
	*Mycoplasma gallisepticum*	vitro	PBMCs (chicken), HEK293T (human), and DF-1 cells (human)	[66]
	*Salmonella*	vitro	MDCK cells (dog) and HeLa cells (human)	[59]
	*Escherichia coli*	vitro	COS-7 cells (monkey)	[60]
	*Streptococcus anginosus*	vitro	Ges-1 (human) and AGS cells (human)	[77]
Fungus	Cryptococcal	vivo	Mice	[29]
		vitro	hCMEC/D3 cells (human) and BMEC cells (human)	[72,73]
Virus	CMV	vitro	Human foreskin fibroblast cell lines (human)	[80]
	IAV	vitro	MDCK cells (dog) and A549 cells (human)	[40,84]
	HCV	vitro	Huh-7 cells (human), Huh-7.5 cells (human), and HEK293 cells (human)	[87,98]
	PRV	vivo	Mice	[41]
		vitro	PK-15 cells (pig) and 3D4/21 cells (pig)	[41]
	PRRSV	vitro	Marc-145 cells (monkey), PAMs (pig), and 293T cells (human)	[42,88]
	CSFV	vitro	PK-15 cells (pig)	[43,90]
	HPV	vitro	HeLa cells (human) and HaCaT cells (human)	[91,92,93]
	ARV	vitro	Vero cells (monkey) and DF-1 cells (chicken)	[94]

Note: species of cellular origin in brackets.
